# Protocatechuic Acid from Pear Inhibits Melanogenesis in Melanoma Cells

**DOI:** 10.3390/ijms18081809

**Published:** 2017-08-21

**Authors:** Xuan T. Truong, Seo-Hee Park, Yu-Geon Lee, Hang Yeon Jeong, Jae-Hak Moon, Tae-Il Jeon

**Affiliations:** 1 Department of Animal Science, Chonnam National University, Gwangju 61186, Korea; trongxuan.vp@gmail.com (X.T.T.); seohee55@jnu.ac.kr (S.-H.P.); ugun2@naver.com (Y.-G.L.); 2 Department of Food Science and Technology, BK21 Plus Program, Chonnam National University, Gwangju 61186, Korea; wjdgkddus@naver.com (H.Y.J.); nutrmoon@chonnam.ac.kr (J.-H.M.)

**Keywords:** hyperpigmentation, melanogenesis, melanoma, pear, protocatechuic acid

## Abstract

Despite the critical role of melanin in the protection of skin against UV radiation, excess production of melanin can lead to hyperpigmentation and skin cancer. Pear fruits are often used in traditional medicine for the treatment of melasma; therefore, we investigated the effects of pear extract (PE) and its component, protocatechuic acid (PCA), on melanogenesis in mouse melanoma cells. We found that PE and PCA significantly suppressed melanin content and cellular tyrosinase activity through a decrease in the expression of melanogenic enzymes and microphthalmia-associated transcription factor (*Mitf*) in α-melanocyte stimulating hormone-stimulated mouse melanoma cells. Moreover, PCA decreased cyclic adenosine monophosphate (cAMP) levels and cAMP-responsive element-binding protein phosphorylation, which downregulated *Mitf* promoter activation and subsequently mediated the inhibition of melanogenesis. These results suggested that pear may be an effective skin lightening agent that targets either a tyrosinase activity or a melanogenic pathway.

## 1. Introduction

Melanin pigment, the primary determinant of skin and hair color in mammals, also protects the skin from injuries related to UV irradiation [1]. However, excessive cutaneousmelanin deposition, which may occur through increases in the number of melanocytes or melanin synthesis, can lead to hyperpigmentation disorders such as melasma, actinic and senile lentigines, and post-inflammatory hyperpigmentation [2,3].

Melanin is synthesized through the complex process of melanogenesis, which occurs within vesicles called melanosomes in melanocytes and is mediated by melanocyte-specific enzymes, such as tyrosinase and tyrosinase-related proteins (TRPs) [4]. Tyrosinase is the rate-limiting enzyme that catalyzes the hydroxylation of tyrosine into dihydroxyphenylalanine (DOPA), followed by the subsequent oxidation of DOPA into dopaquinone. TRP1 and TRP2 are also present in the melanosome and play a critical role in melanin biosynthesis [5].

Melanogenesis is mainly stimulated by α-melanocyte stimulating hormone (α-MSH), which is produced from proopiomelanocortin in response to UV irradiation [6]. α-MSH activates the cyclic adenosine monophosphate (cAMP)-protein kinase A (PKA) pathway through the melanocortin receptor 1 (MC1R), the activation of which upregulates the transcription of microphthalmia-associated transcription factor (MITF), the master regulator in the transcription of genes encoding melanogenic enzymes [4]. Alterations in MITF expression are highly associated with abnormal skin and hair pigmentation [7]. Indeed, it has been reported that the downregulation of MITF expression by tea catechins was responsible for a reduction of melanin synthesis in mouse and human melanoma [8]. Thus, MITF is an attractive molecular target for the potential treatment or prevention of hyperpigmentation disorders.

Asian pear (mostly *Pyrus* spp.) belongs to the Rosaceae family and is one of the most widely consumed fruits in Eastern Asia. It is not only used as a fruit but also as a traditional medicine against cough, diuresis, and melasma [9]. Many investigations have revealed that the presence of polyphenol-rich foods in the diet may be related to a lower risk of hyperpigmentation [3] and various phenolic compounds from pear fruits have been identified in our previous studies [10,11,12]. Therefore, in this study, we investigated the anti-melanogenic effects of pear fruit extract and evaluated the depigmentation mechanisms of the extract and its constituent protocatechuic acid (PCA) in mouse melanoma cells.

## 2. Results and Discussion

### 2.1. Pear Extract (PE) Inhibits Melanin Synthesis in B16F10 Cells

**Figure 1:**
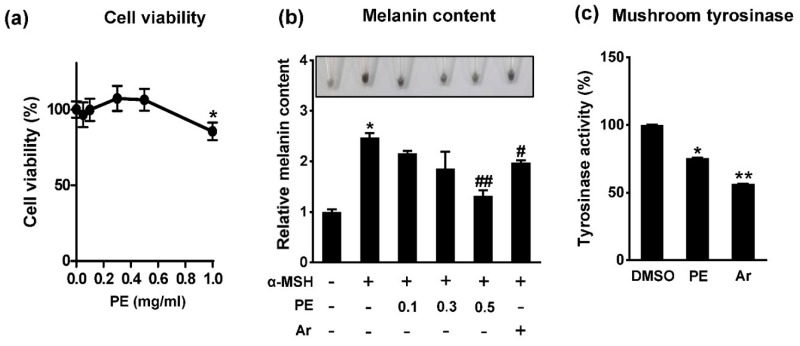
Effects of pear extract (PE) on melanogenesis in mouse melanoma cells. (**a**) B16F10 cells were treated with various concentrations of PE for 72 h. The cell viability was measured by 3-(4,5-dimethylthiazol-2-yl)-2,5-diphenyltetrazolium bromide (MTT) assay. * *p* < 0.05 vs. control dimethyl sulfoxide (DMSO); (**b**) Cells were exposed to 1 μM α-melanocyte stimulating hormone (α-MSH) in presence or absence of PE (mg/mL) or 2 mM arbutin (Ar) for 72 h. Cell pellet images were taken using a digital camera and melanin contents were measured with synthetic melanin as standard. * *p* < 0.05 vs. control; ^#^ *p* < 0.05 vs. α-MSH; ^##^ *p* < 0.01 vs. α-MSH; (**c**) Mushroom tyrosinase activity in the absence or presence of PE (0.5 mg/mL) or arbutin (2 mM). * *p* < 0.05 vs. DMSO; ** *p* < 0.01 vs. DMSO.

To determine the effects of pear extract (PE) on cytotoxicity, B16F10 cells were treated with a range of PE concentrations, from 0.05 to 1 mg/mL, for 72 h and the cell viability was measured using the 3-(4,5-dimethylthiazol-2-yl)-2,5-diphenyltetrazolium bromide (MTT) assay. PE exerted no significant cytotoxicity up to 0.5 mg/mL (Figure 1a). Next, to assess the effect of PE on melanin production in B16F10 cells, cells were stimulated with α-MSH for 72 h in the presence or absence of PE. α-MSH induced an increase in melanin content, but PE treatment decreased the α-MSH-induced melanin increase in a dose-dependent manner, as shown in Figure 1b. The inhibitory effect was superior to that of 2 mM arbutin, which was used as positive control. Several depigmenting agents decrease melanin synthesis in melanocytes through the inhibition of tyrosinase activity [13], and we have previously identified arbutin, which was extracted from PE, as a tyrosinase inhibitor [14]; therefore, the inhibitory activity of PE towards tyrosinase, the key enzyme of melanogenesis, was also evaluated. Although α-MSH-induced melanin production was dramatically inhibited by 0.5 mg/mL PE (Figure 1b), the extract showed only moderate inhibitory effects on tyrosinase activity in comparison with arbutin (Figure 1c), which suggested that mechanisms other than the direct inhibition of tyrosinase activity may be responsible for the anti-melanogenic effect of PE.

### 2.2. PE Inhibits Melanogenic Gene Expression

**Figure 2:**
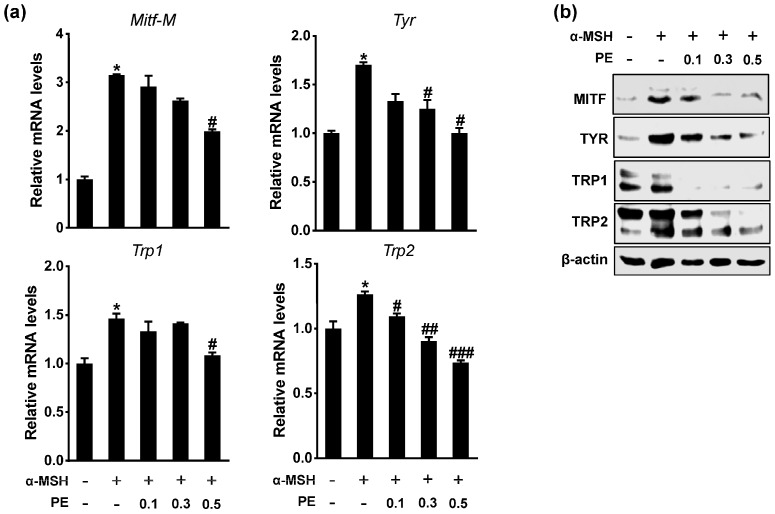
Effects of PE on expression of melanogenic genes. B16F10 cells were exposed to α-MSH in presence or absence of PE (mg/mL) for 24 h. (**a**) mRNA levels for *Mitf-M*, *Tyr*, *Trp1*, and *Trp2* were analyzed by RT-qPCR. * *p* < 0.05 vs. control; ^#^ *p* < 0.05 vs. α-MSH; ^##^ *p* < 0.01 vs. α-MSH; ^###^ *p* < 0.001 vs. α-MSH. Data are mean ± SEM; *n* = 3; (**b**) Cell lysates were analyzed for microphthalmia-associated transcription factor (MITF), tyrosinase (TYR), tyrosinase-related protein 1 (TRP1), and tyrosinase-related protein 2 (TRP2) protein levels by immunoblotting.

To determine the mechanism of action of PE on melanogenesis, we examined the expression levels of key melanogenic genes such as *Mitf*, *tyrosinase* (*Tyr*), *Trp1*, and *Trp2* in B16F10 cells. As shown in Figure 2, α-MSH significantly increased both the mRNA and protein levels of these genes, whereas PE treatment decreased the α-MSH-induced MITF, TYR, TRP1, and TRP2 levels in a dose-dependent manner. MITF, a basic helix-loop-helix leucine zipper transcription factor, is involved in the pigmentation, proliferation, and survival of melanocytes. The regulation of tyrosinase gene expression by MITF is a critical event in melanogenesis [15]. Therefore, our results suggested that PE suppressed melanogenesis via the MITF-mediated decrease of tyrosinase and TRPs expression.

### 2.3. Protocatechuic Acid Inhibits Melanogenesis

Because arbutin is a well-known tyrosinase inhibitor and was reported as a major constituent of PE in our previous paper [10], it may be responsible for the inhibitory effects of PE on melanogenesis (Figure 1c). However, numerous studies [16,17,18] have shown that arbutin did not affect the mRNA expression of MITF; this was consistently found in our observations (Figure S1), which suggested that other components may be involved in the effects of PE. A recent study reported that PCA inhibited melanin production in B16F10 cells, but a mechanism was not determined [19] and we have previously identified PCA as one of the major constituents of PE [10]. In present study, we quantified the contents of arbutin and PCA by high-performance liquid chromatography (HPLC) analysis (Figure S2). Arbutin and PCA contents (mg/100 g fresh weight) in immature pear fruit were 197.33 ± 6.56 and 0.21 ± 0.01, respectively.

**Figure 3:**
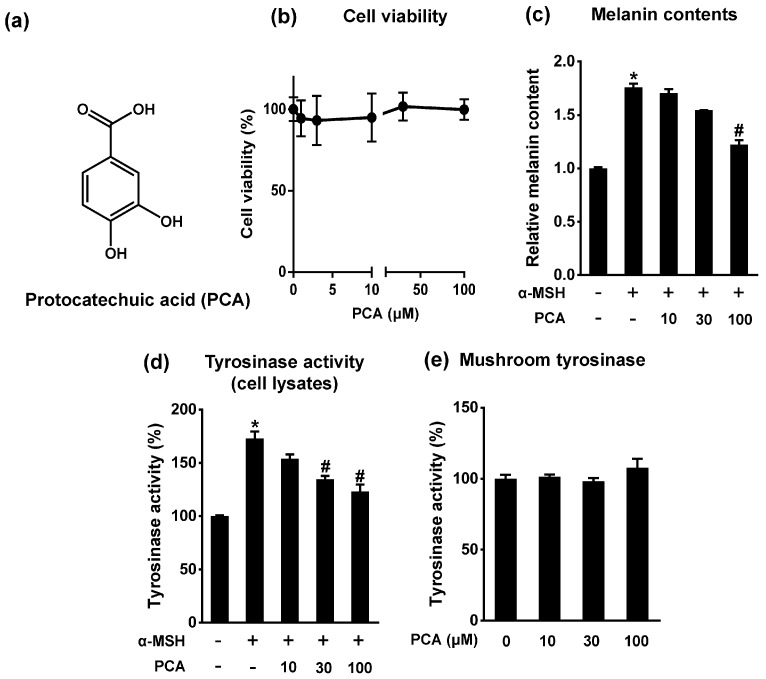
Effects of protocatechuic acid (PCA) on melanin synthesis and tyrosinase activity. (**a**) Structure of PCA; (**b**) B16F10 cells were treated with various concentrations of PCA for 72 h. The cell viability was measured by MTT assay; (**c**) Cells were exposed to 1 μM α-MSH in presence or absence of PCA (μM) for 72 h. Melanin contents were measured with synthetic melanin as standard. * *p* < 0.05 vs. control; ^#^ *p* < 0.05 vs. α-MSH; (**d**) Cells were exposed to 1 μM α-MSH in presence or absence of PCA (μM) for 24 h. Cellular tyrosinase activity. * *p* < 0.05 vs. control; ^#^ *p* < 0.05 vs. α-MSH; (**e**) Mushroom tyrosinase activity in a cell free system. Data are mean ± SEM; *n* = 3.

Therefore, we investigated whether PCA was the active constituent responsible for the antimelanogenic effect of PE in B16F10 cells. PCA markedly suppressed the α-MSH-induced increase in melanin content and cellular tyrosinase activity without affecting cell viability (Figure 3a–d). However, unlike arbutin (Figure 1c), PCA had no direct inhibitory effect on the enzymatic activity of tyrosinase (Figure 3e). There is no doubt that the specific inhibition of tyrosinase activity would be an effective therapy for the reduction of hyperpigmentation, but the use of many tyrosinase inhibitors such as hydroquinone, arbutin, and kojic acid, as depigmenting agents, cause severe adverse effects including skin irritation, genotoxicity, or pigmented contact dermatitis [13]. Our results showed that PCA was not toxic to B16F10 cells and exerted a potent anti-melanogenic effect (Figure 3); therefore, PCA could be a potential non-toxic alternative for hyperpigmentation treatment.

### 2.4. Protocatechuic Acid (PCA) Inhibits Melanogenic Genes through the Downregulation of Microphthalmia-Associated Transcription Factor (MITF) Transcription

**Figure 4:**
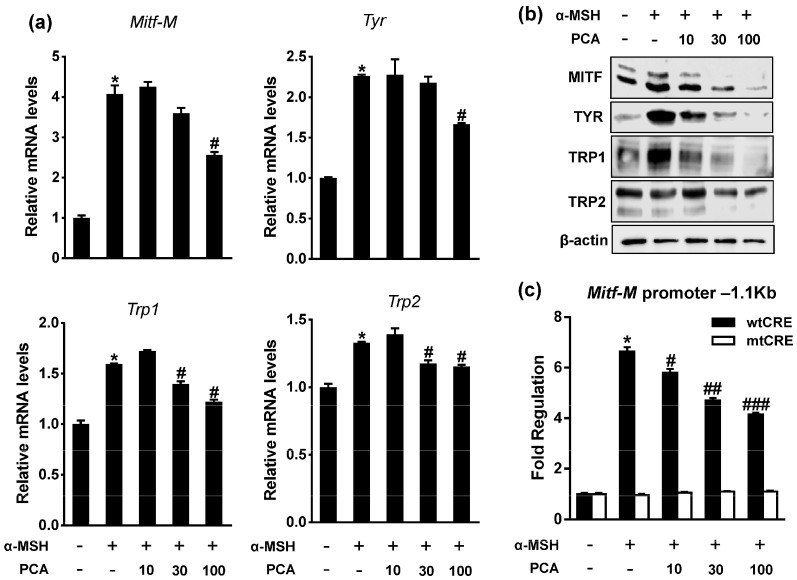
Effects of PCA on expression of melanogenic genes and activation of MITF promoter. B16F10 cells were exposed to α-MSH in presence or absence of PCA (μM) for 24 h. (**a**) mRNA levels for *Mitf-M*, *Tyr*, *Trp1*, and *Trp2* were analyzed by RT-qPCR. * *p* < 0.05 vs. control; ^#^ *p* < 0.05 vs. α-MSH. Data are mean ± SEM; *n* = 3; (**b**) Cell lysates were analyzed for MITF, TYR, TRP1, and TRP2 protein levels by immunoblotting; (**c**) Reporter assays were performed using mouse MITF-M promoter construct. Cells were transfected with wild type (wt) cAMP-responsive element (CRE) or mtCRE luciferase reporters for 24 h before treatment with PCA (μM) and/or α-MSH for 24 h. * *p* < 0.05 vs. control; ^#^ *p* < 0.05 vs. α-MSH; ^##^ *p* < 0.01 vs. α-MSH; ^###^ *p* < 0.005 vs. α-MSH. Data are mean ± SEM; *n* = 3.

Next, we examined whether PCA inhibited the expression of melanogenic genes. Similar to the effects produced by PE, PCA treatment also significantly decreased the mRNA and protein levels of the α-MSH-induced melanogenic genes, including *Mitf*, *Tyr*, *Trp1*, and *Trp2*, in B16F10 cells (Figure 4a,b). Moreover, PE and PCA inhibit melanin contents and mRNA and protein expression of *MITF* and *TYR* in human melanoma SK-MEL-28 cells (Figure S3). The MITF gene has multiple promoters and at least nine promoter-exon units direct the transcription initiation of specific MITF isoforms by alternative splicing [2]. The MITF-M promoter that is located nearest to the common downstream exons is selectively expressed in melanocytes and is targeted by several transcriptional factors including paired box gene 3, cAMP-responsive element-binding protein (CREB), SRY (sex-determining region Y)-box 10 (SOX10), lymphoid enhancer-binding factor 1, and MITF itself [7].

Because both PE and PCA significantly inhibited the expression of *Mitf* mRNA, we examined whether PCA suppressed *Mitf* expression through transcriptional regulation. To test this, B16F10 cells were transiently transfected with luciferase reporter plasmids containing wild type MITF-M promoter (melanocyte-specific isoform) sequences. α-MSH upregulated MITF promoter activity, whereas PCA treatment significantly suppressed the effect of α-MSH in a dose-dependent manner (Figure 4c). Although the activation of the MITF promoter is regulated by several transcription factors, it is classically activated by a conserved cAMP-responsive element (CRE) in response to α-MSH signaling [20]. As shown in Figure 4c, the deletion of the CRE in the MITF promoter (mtCRE) markedly reduced the stimulation of activity in response to α-MSH and completely blocked the inhibitory effect of PCA, which indicated that the CRE motif was essential for the PCA downregulation of MITF transcription.

### 2.5. PCA Downregulates Cyclic Adenosine Monophosphate (cAMP)/cAMP-Responsive Element Binding Protein (CREB) Signaling Pathway

**Figure 5:**
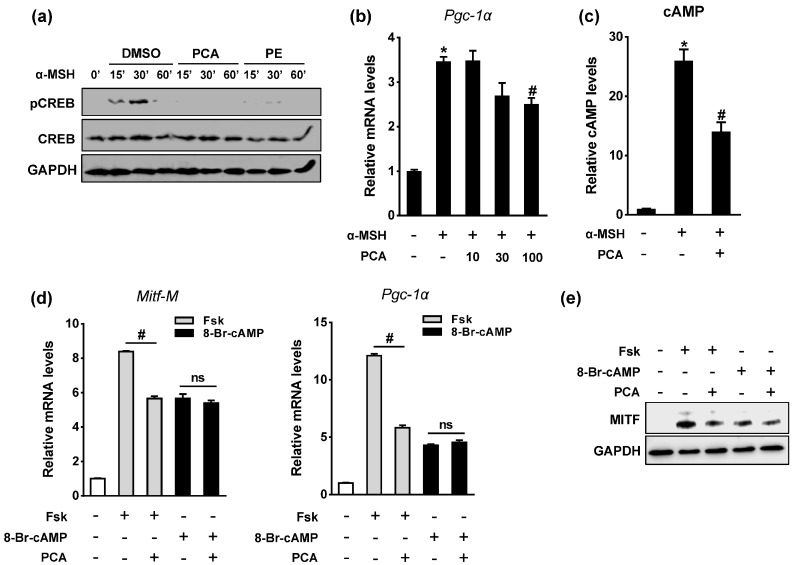
Effects of PCA on CREB phosphorylation and intracellular cAMP level. B16F10 cells were pretreated with 100 μM PCA for 24 h prior to 1 μM α-MSH treatment for indicated time. (**a**) Cell lysates were analyzed for pCREB, CREB, and GAPDH protein levels by immunoblotting; (**b**) mRNA levels for *Pgc-1α* were analyzed by RT-qPCR. * *p* < 0.05 vs. control; ^#^ *p* < 0.05 vs. α-MSH; (**c**) Cells were pretreated with 100 μM PCA for 24 h, and then exposed to 1 μM α-MSH for 30 min. Intracellular cAMP concentration was measured by immunoassay as described in Materials and methods. * *p* < 0.05 vs. control; ^#^ *p* < 0.05 vs. α-MSH; (**d**) Cells were exposed to 20 μM forskolin (Fsk) or 100 μM 8-Br-cAMP in presence or absence of PCA (100 μM) for 24 h. mRNA levels for *Mitf-M* and *Pgc-1α*. ^#^ *p* < 0.05 vs. Fsk; ns, not significant. Data are mean ± SEM; *n* = 3; (**e**) Protein level for MITF.

The binding of α-MSH to MC1R in melanocytes induces the activation of adenylyl cyclase (AC), which is followed by an increase in cAMP levels. The cAMP pathway leads to phosphorylation of the CREB transcription factor, which in turn stimulates MITF-M promoter activation [4]. Thus, to identify the signaling pathways that are regulated by PCA in melanogenesis, we determined whether PCA regulated CREB activation in response to α-MSH. The treatment of B16F10 cells with α-MSH increased CREB phosphorylation, but either PCA or PE completely abrogated it (Figure 5a). α-MSH and cAMP signaling also induced expression of the transcriptional coactivator PPAR-γ coactivator (PGC)-1α in melanocytes, thereby enhancing the transcriptional activation of MITF by CREB and SOX10, which subsequently mediated the induction of melanogenesis [21]. As shown in Figure 5b, α-MSH-induced *Pgc-1α* expression was also significantly decreased by PCA. Because this effect could be mediated by cAMP, we measured the levels of cAMP in B16F10 cells. PCA treatment significantly decreased intracellular cAMP levels in α-MSH-stimulated conditions (Figure 5c), which suggested that the reduction in cAMP accumulation by PCA resulted in the deactivation of CREB and PGC-1α.

To further elucidate the mechanism by which PCA suppressed the α-MSH-induced expression of *Mitf* and *Pgc-1α*, we utilized forskolin and 8-Br-cAMP, which mimic the action of α-MSH in melanogenesis by bypassing MC1R and G proteins. Forskolin is a direct activator of AC and 8-Br-cAMP is a cell-permeable analog of cAMP. Interestingly, PCA decreased the forskolin-induced expression of *Mitf* and *Pgc-1α*, but did not affect the stimulation by 8-Br-cAMP. This indicated that PCA may specifically target AC rather than the downstream effectors of cAMP (Figure 5d,e). Collectively, these results suggested that PCA suppressed the α-MSH-induced MITF transcription via the downregulation of cAMP-mediated CREB activation.

**Figure 6:**
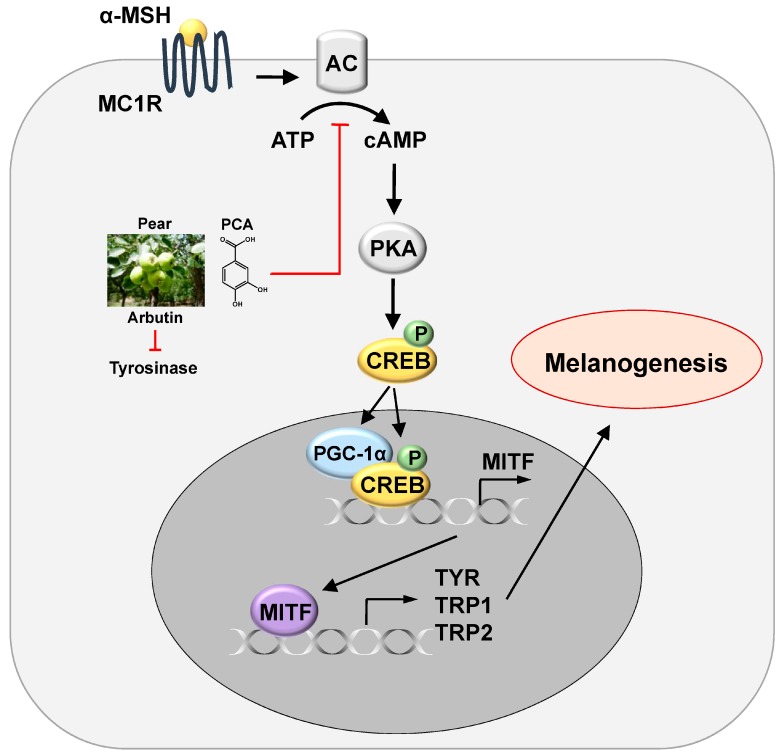
A proposed mechanism for inhibitory effect of PE and its components (PCA and arbutin) on melanogenesis. Arrows indicate positive regulation while T-bars denote inhibitory effects.

Our results indicated that PE suppressed α-MSH-stimulated melanin synthesis and this inhibitory effect was correlated with either a decrease in the enzymatic activity of tyrosinase, probably owing to the presence of arbutin, or a decrease in MITF expression via the reduction of cAMP production, probably owing to presence of PCA (Figure 6).

## 3. Materials and Methods

### 3.1. Materials and Cell Culture

Pear (*Pyrus pyrifolia* Nakai cv. Chuhwangbae) was cultivated in Naju and its immature fruit was collected in May 2015 after 35 days of florescence. The samples were certified by Wol-Soo Kim (Laboratory of Pomology, College of Agriculture and Life Science, Chonnam National University, South Korea). Arbutin (A4256), PCA (37580), α-MSH, melanin, forskolin, 8-bromoadenosine 3′,5′-cyclic monophosphate (8-Br-cAMP), and 3-(4,5-dimethylthiazol-2-yl)-2,5-diphenyltetrazolium bromide (MTT) were purchased from Sigma-Aldrich Chemical Co. (St. Louis, MO, USA). B16F10 and SK-MEL-28 cells were purchased from American Type Culture Collection (ATCC, Manassas, VA, USA) and maintained in Dulbecco’s modified Eagle’s medium supplemented with 10% heat-inactivated fetal bovine serum and 1% antibiotics (100 U/mL penicillin/streptomycin) in an atmosphere of 5% CO_2_ at 37 °C. All cell culture reagents were obtained from Gibco BRL (Grand Island, NY, USA).

### 3.2. Sample Preparation

Immature pear fruit (1 kg, fresh weight) was homogenized with 60% ethanol (4 L) by using a homogenizer (T50 digital ULTRA-TURRAX, IKA, Staufen, Germany). The homogenate was kept at 25 °C for 24 h and filtered under vacuum through no. 2 filter paper (Whatman, Maidstone, UK). The filtrates were concentrated, lyophilized, and stored at −20 °C until used.

### 3.3. Determination of Arbutin and PCA Contents in Immature Pear Fruit

Immature pear fruit extract (1 g fresh weight equivalent) was dissolved in 50% methanol (1 mL) and filtered through a 0.45 μm Millipore membrane filter. The dissolved solution was subjected to Octadecyl-silica (ODS)-HPLC analysis. Arbutin and PCA were separated on an ODS column (UG120, 4.6 mm internal diameter (ID) × 250 mm, 5 μm, Shiseido, Tokyo, Japan). The mobile phase was composed of H_2_O/acetic acid (98:2, *v*/*v*, eluent A) and MeOH/H_2_O (60:40, *v*/*v*, eluent B). The gradient program used was as follows: started at 100% A and increased to 100% B linearly over 45 min. The column temperature was maintained at 40 °C and the flow rate was 1 mL/min. Arbutin and PCA were monitored at 280 and 254 nm, respectively, using a photodiode array detector (SPD-M20D; Shimadzu, Kyoto, Japan). Arbutin and PCA contents in each sample were quantified by the chromatographic peak area of external standards. The calibration curves were plotted in the concentration range of 0.01–10 μg.

### 3.4. Cell Viability

The cells were seeded in 96-well plates, treated with various concentrations of PE or PCA for 72 h, and then incubated with MTT for 2 h. After the incubation, the cells were thoroughly washed with phosphate-buffered saline (PBS) and the insoluble formazan product was dissolved in dimethyl sulfoxide (DMSO). The absorbance of each well was measured at 560 nm by using a microplate reader (Biotek, Winooski, VT, USA). The absorbance of 0.1% DMSO-treated control cells was considered to represent 100% viability.

### 3.5. Determination of Melanin Content

The cells were treated with α-MSH (1 μM) in the presence or absence of PE, PCA, or arbutin (positive control) for 72 h, trypsinized and centrifuged at 1000× *g* for 5 min at 4 °C. The cell pellets were photographed and then solubilized in 1 N NaOH containing 10% DMSO at 80 °C for 1 h. The melanin content was calculated by the comparison of the absorbance at 405 nm with those of a standard curve of synthetic melanin.

### 3.6. Measurement of Tyrosinase Activity

To measure the tyrosinase activity in a cell free system, 1250 U/mL of mushroom tyrosinase solution (20 μL) was added to a mixture of 50 mM sodium phosphate buffer, pH 6.8 (100 μL), the desired sample (5 μL), and 5 mM l-tyrosine (125 μL). After incubation for 5 min at 25 °C, the production of dopachrome was determined by monitoring the change in absorbance at 475 nm every 20 s for 30 min.

To measure cellular tyrosinase activity, the cells were first treated with PCA for 24 h and then α-MSH (1 μM) was added. After incubation for 24 h, the cells were washed with PBS and centrifuged at 1000× *g* for 5 min at 4 °C. The cell pellets were resuspended in radioimmunoprecipitation assay (RIPA) buffer and centrifuged at 20,000× *g* for 20 min at 4 °C. The supernatant was used as a crude enzyme solution. The crude enzyme solution (20 μL; equivalent to 100 μg protein) was added to a mixture of 50 mM sodium phosphate buffer, pH 6.8 (100 μL) and 5 mM l-tyrosine (30 μL). After incubation for 3 h at 37 °C, the absorbance of the solution was measured at 475 nm.

### 3.7. RNA Analysis

Total RNA was isolated using TRIzol™ (Invitrogen, Carlsbad, CA, USA), cDNA was synthesized using ReverTra Ace^®^ qPCR RT kit (Toyobo, Osaka, Japan), and quantitative PCR was performed using a Mx3000P qPCR System (Agilent Technologies, Santa Clara, CA, USA). The primer sequences used in this study are shown in Table S1. mRNA levels were normalized to the expression of mouse ribosomal protein, Large, P0 (Rplp0), and the data were calculated by the comparative threshold cycle method.

### 3.8. Immunoblotting

The cells were washed with PBS and centrifuged at 1000× *g* for 5 min at 4 °C. The obtained pellets were resuspended in RIPA buffer with protease and proteasome inhibitors, incubated on ice for 20 min, sonicated, and centrifuged at 20,000× *g* for 20 min at 4 °C. The supernatants were separated using 8% or 10% SDS-PAGE and then transferred to nitrocellulose membranes. The membranes were incubated overnight at 4 °C with primary antibodies against MITF, GAPDH (Thermo Fisher Scientific, Rockford, IL, USA), tyrosinase, TRP1, TRP2, β-actin (Santa Cruz Biotechnology, Santa Cruz, CA, USA), cAMP-responsive element binding protein (CREB), or pCREB (Cell Signaling Technology, Danvers, MA, USA), which was followed by incubation with the appropriate secondary antibodies (Thermo Fisher Scientific, Rockford, IL, USA). The blots were developed by using the EZ-Western Lumi Femto™ western blot detection kit (Daeil Lab Service, Seoul, Korea).

### 3.9. Luciferase Reporter Assay

The mouse MITF-M promoter construct (−1143 to +48, wtCRE) was amplified via PCR using mouse genomic DNA as template, and cloned into the pGL3-basic firefly luciferase reporter vector (Promega, Madison, WI, USA). The mtCRE was constructed by deleting the CRE motif (−153/−146, 5′-TGACGTCA-3′) by using the site-directed mutagenesis kit (Agilent Technologies, Santa Clara, CA, USA). All constructs were verified via DNA sequencing. The cells were seeded in 24-well plates and transfected for 24 h with pGL3-Mitf-M (wtCRE or mtCRE) luciferase reporter, using Lipofectamine^®^ 2000 reagent (Invitrogen, Carlsbad, CA, USA), before treatment with α-MSH and/or PCA for a further 24 h. The cells were harvested and assayed using the Nano-Glo^®^ Dual-Luciferase^®^ Reporter Assay System (Promega, Madison, WI, USA). The pNL1.1 luciferase vector (Promega, Madison, WI, USA) was used as a normalization control.

### 3.10. cAMP Determination

The cells were pretreated with PCA for 24 h, exposed to α-MSH for 30 min, washed in PBS, and lysed in 0.1 M HCl. After centrifugation, the cAMP in the supernatant was measured by a cAMP assay kit (BioVision, Milpitas, CA, USA).

### 3.11. Statistical Analysis

All experiments were performed a minimum of three times. The data were presented as the mean ± SEM. The differences between the means of the individual groups were assessed using Student’s *t*-test or one-way analysis of variance (ANOVA); differences were considered significant at *p* < 0.05. The statistical software package Prism 6.0 (GraphPad Software, La Jolla, CA, USA) was used for these analyses.

## 4. Conclusions

Pear fruit and its constituent PCA effectively inhibit cellular tyrosinase activity and expression through cAMP/CREB/MITF-mediated mechanism, which contributes to reduction in α-MSH-stimulated melanogenesis in mouse and human melanoma. These effects of pear may help to prevent or treat hyperpigmentation.

## Supplementary Materials

Supplementary materials can be found at www.mdpi.com/1422-0067/18/8/1809/s1.
