# Supplementary material for: Protocatechuic Acid from Pear Inhibits Melanogenesis in Melanoma Cells
Source: Int J Mol Sci. 2017 Aug 21;18(8):1809. doi: 10.3390/ijms18081809 (PMC5578196; doi:10.3390/ijms18081809)
Supplement: Supplementary file 1 [file ijms-18-01809-s001.pdf]

Supplementary Information

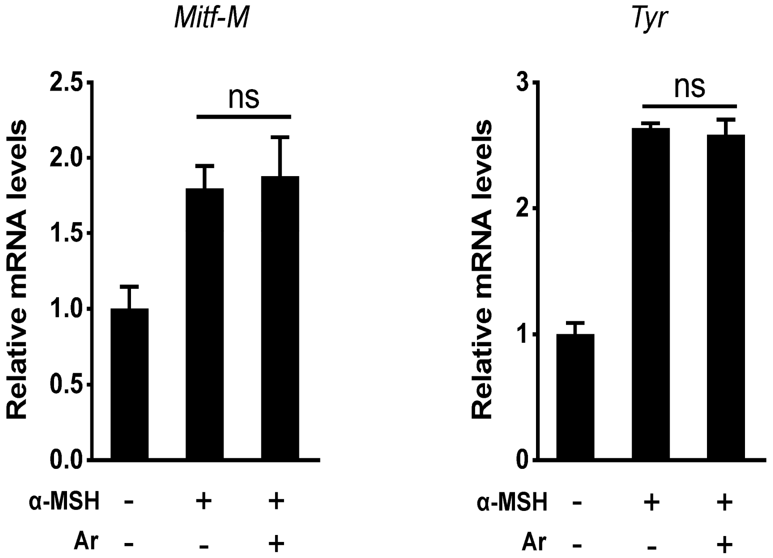

Supplementary Figure 1. Effect of arbutin on the expression of melanogenic genes in B16F10 cells. Cells were exposed to 1 μM α-MSH in presence or absence of 2 mM arbutin (Ar) for 24 h. ns, not significant. Data are mean ± SEM; n=3.

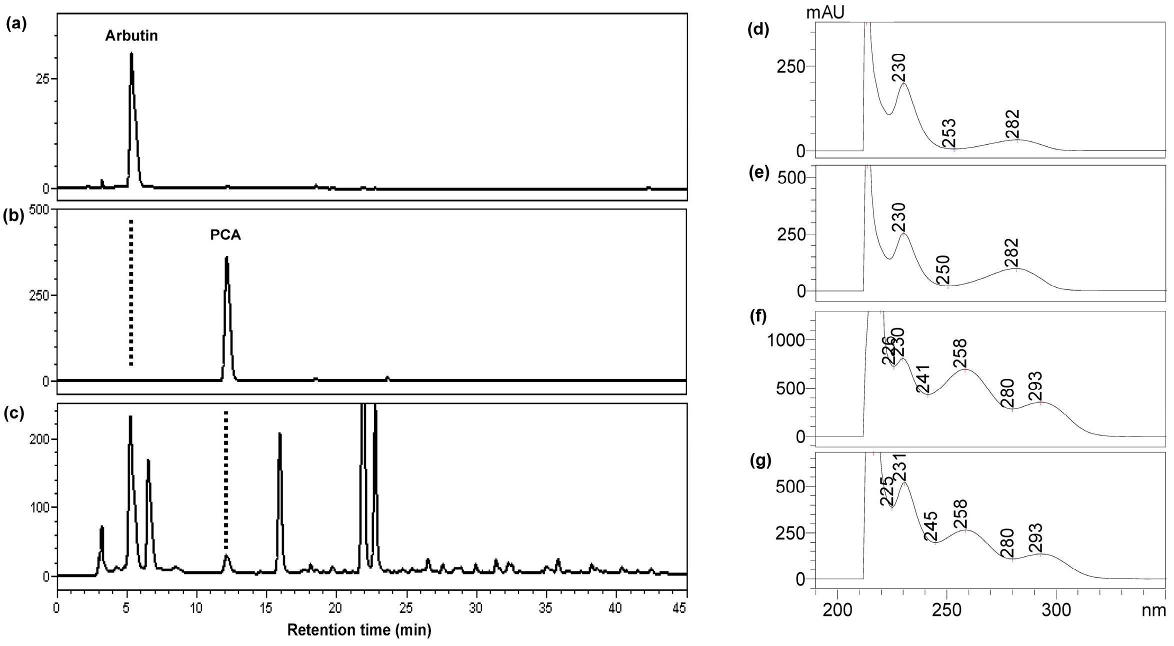

Supplementary Figure 2. HPLC chromatograms and PDA UV/VIS spectrums of arbutin, PCA, and immature pear fruit extract. (a) Arbutin standard. (b) PCA standard. (c) immature pear fruit extract. The peaks of arbutin and PCA on the HPLC chromatograms were detected with a retention time (t_R) of 5.4 and 12.3 min, respectively. (d) UV/VIS spectrum of arbutin standard. (e) UV/VIS spectrum of arbutin in immature pear fruit extract. (f) UV/VIS spectrum of PCA standard. (g) UV/VIS spectrum of PCA in immature pear fruit extract.

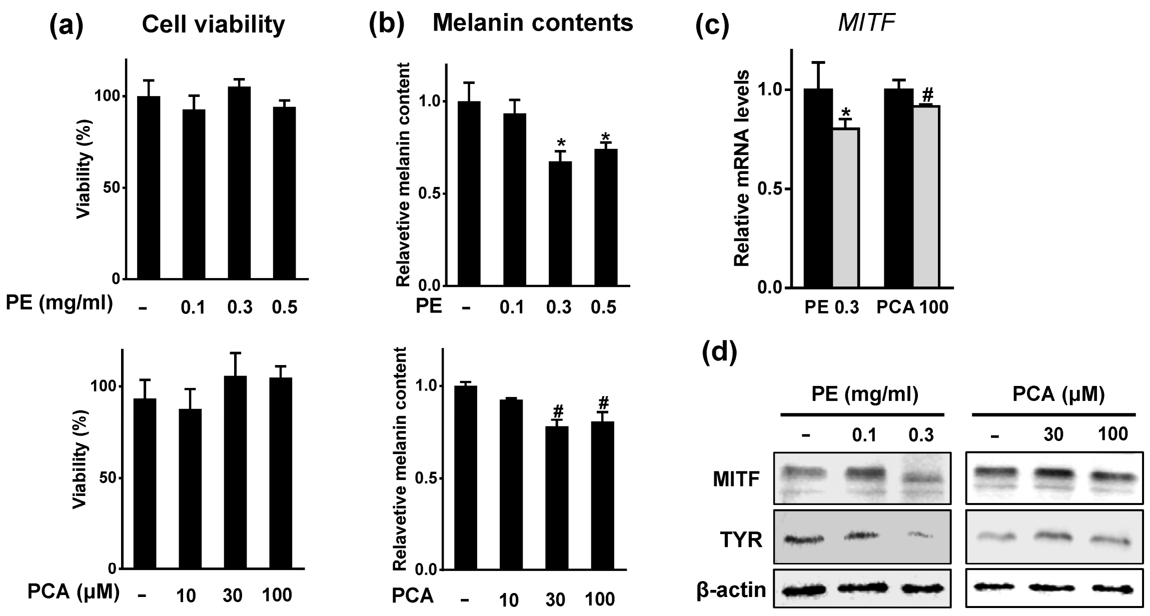

Supplementary Figure 3. Effect of PE and PCA on melanogenesis in human melanoma SK-MEL-28 cells. Cells were exposed to PE or PCA for 48 h. (a) The cell viability was measured by MTT assay. (b) Melanin contents were measured with synthetic melanin as standard. (c and d) mRNA and protein levels were analyzed by RT-qPCR and immunoblotting, respectively. **P* <0.05 vs. PE control; #*P* <0.05 vs. PCA control. Data are mean ± SEM; n=3.

Table 1. Primers used in this study

Genes	Forward	Reverse
**RT-qPCR**		
Mitf-M	GCCTGAAACCTTGCTATGCTGGAA	AAGGTACTGCTTTACCTGGTGCCT
Tyr	CTCTGGGCTTAGCAGTAGGC	GCAAGCTGTGGTAGTCGTCT
Trp1	TGGGGATGTGGATTTCTCTC	AGGGAGAAAGAAGGCTCCTG
Trp2	AGGTACCATCTGTTGTGGCTGGAA	AGTTCCGACTAATCAGCGTTGGGT
Pgc-1α	AACCACACCCACAGGATCAGA	TCTTCGCTTTATTGCTCCATGA
Rplp0	GTGCTGATGGGCAAGAAC	AGGTCCTCCTTGGTGAAC
MITF-M	CCGTCTCTCACTGGATTGGT	TACTTGGTGGGGTTTTCGAG
TYR	TTGTACTGCCTGCTGTGGAG	CAGGAACCTCTGCCTGAAAG
**Cloning**		
wtCRE	GAAGATCTCTGAAAATGCCTGCGAACCT	CCCAAGCTTGACCGGCATAAAGAAGGCAC
mtCRE	AAAAAGGTTCTTTTATATCTATGAAAAAAA<br>GCAAGCCAGCAGGGAAACTG	CAGTTTCCCTGCTGGCTTGCTTTTTTTCATA<br>GATATAAAAGAACCTTTTT
